# Moderate Prenatal Cadmium Exposure and Adverse Birth Outcomes: a Role for Sex‐Specific Differences?

**DOI:** 10.1111/ppe.12318

**Published:** 2016-10-25

**Authors:** Caroline M. Taylor, Jean Golding, Alan M. Emond

**Affiliations:** ^1^ Centre for Child and Adolescent HealthSchool of Social and Community MedicineUniversity of BristolBristolUK

**Keywords:** ALSPAC, cadmium, pregnancy, environment, smoking, child, birth outcomes, heavy metals

## Abstract

**Background/Aim:**

Studies on the effects of moderate prenatal exposure to cadmium (Cd) on birth outcomes have been contradictory and it has been suggested that effects may be partly masked by sex‐specific effects. Our aim was to examine the association of Cd exposure in a large group of pregnant women with birth outcomes in the whole group of participants and by sex.

**Methods:**

Pregnant women were enrolled in the Avon Longitudinal Study of Parents and Children (ALSPAC). Whole blood samples for singleton pregnancies with a live birth were analysed for Cd (*n* = 4191). Data collected on the infants included anthropometric variables and gestational age at delivery. Data were analysed using SPSS v18.

**Results:**

There were adverse associations of maternal blood Cd level with birthweight (unstandardized B coefficient −62.7 g, 95% CI −107.0, −18.4) and crown–heel length (−0.28 cm, 95% CI −0.48, −0.07) in adjusted regression models. On stratification by sex, maternal blood Cd level was adversely associated with birthweight (−87.1 g, 95% CI −144.8, −29.4), head circumference (−0.22 cm, 95% CI −0.39, −0.04), and crown–heel length (−0.44 cm, 95% CI −0.71, −0.18) in girls but not in boys in adjusted regression models.

**Conclusion:**

In these pregnant women with moderate prenatal Cd exposure there evidence of adverse associations with birth anthropometry variables in the whole group. However, there was evidence of associations with anthropometric variables in girls that were not evident in boys. Sex‐specific effects require further investigation in large cohorts as a possible contributor to the lack of associations generally found in mixed‐sex studies.

Cadmium (Cd) is a non‐essential cumulative toxic metal that is widespread in the environment. Occupational exposure is generally associated with battery manufacture and recycling, and non‐occupational exposure arises through fossil fuel combustion, waste incineration, and manufacturing processes including those for cement, iron, and steel. Smoking tobacco, however, is the most important source of Cd in the general population: cigarettes contain about 0.5–1.5 μg Cd/cigarette, with 20–50% of this transferred directly to the circulation.^1^ For non‐smokers, the primary source of exposure is through diet, with grains and grain products, vegetables and vegetable products, and starchy roots and tubers making the greatest contributions to total ingestion (27%, 16%, and 13%, respectively).^2^ Cd absorption may be dependent on micronutrient status, particularly calcium, zinc, and iron.^3^

Adverse health effects from Cd exposure include kidney, breast, prostate, and lung cancer; cardiovascular disease; and osteoporosis. These diseases may be related to the role of Cd as an endocrine disruptor through interaction with hormone signalling pathways.^4^ Its effects on birth outcomes, however, particularly at moderate exposures, are not well understood and in this regard Cd has received far less research attention than its near neighbours in the periodic table, lead and mercury, The placenta is thought to be at least a partial barrier to Cd transfer to the fetus,^5^, ^6^ but determination of ratios of Cd in cord blood : maternal blood have left this in doubt (ratios ranging from 0.24^7^ to 1.08,^8^ with others finding intermediate values^9^, ^10^). Studies with moderate maternal blood cadmium levels (B‐Cd; below a suggested reference value of 1 μg/L^11^) and adjustment for smoking have generally found no associations with birthweight, birth length, head circumference or preterm delivery,^8^, ^12^, ^13^ but other studies have found negative associations particularly with birthweight^14^, ^15^, ^16^ (Table S1). Even where exposure levels are higher and effects could potentially be more evident, again some studies have found no associations with birth outcomes,^10^, ^17^ while others have found adverse associations.^18^ It has recently been suggested that adverse events associated with maternal Cd exposure are sex‐specific, with effects on birthweight and birth length being more severe in or confined to girls^8^, ^19^, ^20^, ^21^ and that this might mask effects in mixed‐sex studies.

The aims of this study were: (1) to evaluate the effect of moderate prenatal exposure to Cd on a range of birth outcomes (birthweight, head circumference, crown–heel length, preterm delivery, and low birthweight) independently of active and passive smoking status in a large cohort of pregnant women, (2) to evaluate sex‐specific effects of maternal Cd exposure on these birth outcomes.

## Methods

### The ALSPAC study

The sample was derived from the ALSPAC study, a population‐based study investigating environmental and genetic influences on the health, behaviour, and development of children. This database provided an opportunity to include a large number of participants and includes a wide range of social and demographic information to enable the most appropriate selection of covariates. All pregnant women in the former Avon Health Authority with an expected delivery date between April 1991 and December 1992 were eligible for the study; 14 541 pregnant women were enrolled, resulting in a cohort of 14 062 live births. The social and demographic characteristics of this cohort were similar to those found in UK national census surveys. Further details of ALSPAC are available at www.bris.ac.uk/alspac and the study website contains details of all the data that are available through a fully searchable data dictionary (http://www.bris.ac.uk/alspac/researchers/data-access/data-dictionary). Ethics approval for the study was obtained from the ALSPAC Ethics and Law Committee and the Local Research Ethics Committees.

### Collection, storage, and analysis of blood samples

Whole blood samples were collected in acid‐washed vacutainers (Becton and Dickinson, Oxford, UK) by midwives as early as possible in pregnancy (median of 11 weeks’ gestation (IQR 9–13 weeks) with a mode of 10 weeks). Whole blood samples were stored in the original tube at 4°C at the collection site before being transferred to the central Bristol laboratory within 1–4 days. Samples were at ambient temperature during transfer (up to 3 h). They were then stored at 4°C until analysis. Details of the analysis have been reported.^22^ In brief, inductively coupled plasma mass spectrometry in standard mode (R. Jones, Centers for Disease Control and Prevention (CDC), Bethesda, MD, USA CDC; Method 3009.1) was used to measure blood levels of Cd with appropriate quality controls. The analyses were completed for 4484 women for Cd. 1119 samples had a Cd level below the limit of detection (0.2 μg/L): these were assigned a value of 0.14 μg/L (LOD/√2) to reflect the log‐normal distribution.^23^

### Pregnancy outcomes

Newborn head circumference and crown–heel length were measured by trained study staff where the mother gave permission, or if these data were missing, the values were extracted from the medical records by trained study staff. Birthweight was derived from obstetric data and from central birth notification data: where values disagreed by <100 g then the lowest value was accepted; if the values disagreed by >100 g then the value was coded as missing. Study staff were blinded to the maternal blood Cd. Length of gestation was based on last menstrual period date, ultrasound assessment or other clinical indicators. Where there was conflict of ≥14 days between the maternal report and ultrasound assessment, an experienced obstetrician reviewed the clinical records and made a best estimate. Low birthweight (LBW) was defined as birthweight <2500 g and preterm birth as <37 weeks gestation.

### Questionnaires

The mothers received four postal self‐completion questionnaires during pregnancy. The questionnaires are available from the study website (http://www.bristol.ac.uk/alspac/researchers/questionnaires/).

Information collected on environmental and life style factors from questionnaires during pregnancy included data on age, parity, social class, highest educational qualification, alcohol consumption, cigarette smoking, etc. Data on cigarette smoking were taken from a questionnaire completed at enrolment at gestation ≤14 weeks in response to a question on the number of cigarettes smoked in the previous 2 weeks (categorized as yes/no) and on whether the partner smoked (yes/no). If gestation was >14 weeks at enrolment, the questionnaire was completed 4–7 weeks after enrolment.

### Statistical analysis

Participants were excluded from the study if they did not have a singleton live birth (see Figure S1 for study flow chart). Statistical analysis was done with SPSS version 23 (IBM Corp., Chicago, IL, USA). Values are reported as mean±SD. Chi‐square tests were used to analyse differences in categorical data, and ANOVA was used to compare continuous values by tertiles of B‐Cd.

The main analyses were conducted for term deliveries only (≥37 weeks). Additional analyses were conducted for all gestational ages (results shown in supporting information Tables). Univariate and multivariable linear regression models were used to examine the relationship of B‐Cd with birthweight, head circumference, and crown–heel length. Logistic regression analysis was used to examine the effect of the binary outcome variable LBW (<2500 g) by tertiles of maternal B‐Cd (the binary outcome preterm birth <37 weeks was also included in analyses of the group including all gestational ages). Model 1 included the following confounders: maternal age, maternal height, maternal pre‐pregnancy body mass index, maternal educational attainment, parity (0 versus ≥1), alcohol consumption (number of units per week), and sex of the baby. These factors were chosen as being consistently associated with birthweight, length of gestation, and fetal growth retardation as well as other measures including low birthweight, preterm delivery, and small‐for‐gestational‐age (SGA) at birth as well as B‐Cd. We did not include maternal ethnicity in any of the models because of the low numbers of ethnic minority participants included in the sample (non‐white 2.4%). Model 2 also included maternal smoking (yes/no); partner smoking (yes/no) was added for model 3. Gestational age was not included as a confounder in the association between prenatal exposure and birth outcome as it is considered to be a collider in the association between environmental exposure and birth outcomes, and could therefore mask associations.^24^ To address this we excluded preterm births from the main analyses; however, the effect of gestational age was assessed as an additional confounder to model 3 for the whole group (results shown in supporting information Tables).

We undertook three further analyses. (1) Since there was collinearity between smoking and B‐Cd, models 1–3 for all births were repeated after stratification for smokers and non‐smokers. (2) The mediating effect of Cd in the association between smoking and anthropometric outcomes was assessed with models of the associations with and without adjustment for the maternal B‐Cd level. (3) Sex‐specific effects of maternal B‐Cd were evaluated by repeating models 1–3 for all births after stratification by the sex of the offspring.

Regression diagnostics were used to check that the models fitted the observed data well and to identify any cases that had undue influence on the model.

## Results

### Characteristics of mothers and offspring

There were 4191 singleton livebirths included in the study, of which 3838 were a term birth and were included in the main analyses. Mothers with data on B‐Cd were broadly similar to those without, except that they had a higher educational attainment and were slight older.^22^ The characteristics of the mothers are shown in Table S2.

The mean maternal B‐Cd level was 0.56 ± 0.62 (range 0.14–6.30) (median 0.29, interquartile range 0.14–0.68) μg/L. As reported previously, the mean B‐Cd level was similar to those reported in other developed countries.^25^ The mean B‐Cd level in smokers was 77% greater than in non‐smokers and increased with the number of cigarettes smoked (Table S3). Levels were also greater in mothers whose partners smoked (Table S3) (22% greater for non‐smoking mothers whose partners smoked and 79% greater if both mother and partner were smokers compared with both non‐smokers; *P* < 0.001).

### Regression models for B‐Cd and birth outcomes

In univariate analyses with tertiles of B‐Cd, there was strong evidence for negative associations of maternal B‐Cd with birthweight, head circumference, and crown–heel length (all *P* ≤ 0.001; Table 1). The unadjusted models predicted reductions of 133 g in birthweight, 2 mm in head circumference, and 6 mm in crown–heel length with each 1 μg/L increase in maternal B‐Cd (Table 2). These associations were maintained with adjustment for maternal educational attainment, age, parity, pre‐pregnancy BMI, height, and alcohol intake, and sex of the baby (model 1). When maternal smoking was included, the strong associations for birthweight and crown–heel length were maintained (model 2), but the association for head circumference was slightly attenuated. Additional adjustment for partner smoking status did not alter the outcomes of model 2 greatly (slight attenuation) (model 3). The results were broadly similar when preterm births were included (Tables S4 and S5). Adjustment for gestational age attenuated the associations of maternal B‐Cd with the anthropometric variables (Table S5).

**Table 1:** Birth outcomes by maternal B‐Cd tertile in ALSPAC

	Tertile of maternal B‐Cd			*P* value
	T1	T2	T3	
Mean maternal B‐Cd (μg/L)	0.15 ± 0.02 (range 0.14–0.21, *n* = 1223)	0.30 ± 0.06 (range 0.22–0.44, *n* = 1336)	1.22 ± 0.68 (range 0.45–6.30, *n* = 1269)	
Birthweight (g)	3539 ± 482, *n* = 1212	3532 ± 481, *n* = 1322	3379 ± 482, *n* = 1254	<0.001
Head circumference (cm)	35.03 ± 1.38, *n* = 1048	35.02 ± 1.28, *n* = 1151	34.76 ± 1.30, *n* = 1114	<0.001
Crown–heel length (cm)	51.07 ± 2.10, *n* = 1035	51.14 ± 2.10, *n* = 1139	50.50 ± 2.16, *n* = 1103	<0.001
LBW				
Yes	22 (1.8%)	18 (1.4%)	29 (2.3%)	0.196
No	1190 (98.2%)	1304 (98.6%)	1225 (97.7%)	

Live singleton births, gestational age ≥37 weeks.

ANOVA or chi‐squared test.

**Table 2:** Associations of maternal B‐Cd with birth outcomes in ALSPAC (linear regressions)

	*n*	*R* ^2^	Unstandardized B coefficient (95% CI)
Birthweight			
Unadjusted	3778	0.029	−133.4 (−158.2, −108.6)
Model 1	2709	0.149	−116.0 (−146.6, −85.4)
Model 2	2707	0.152	−72.0 (−114.0, −30.1)
Model 3	2578	0.150	−62.7 (−107.0, −18.4)
Head circumference			
Unadjusted	3313	0.011	−0.22 (−0.29, −0.15)
Model 1	2400	0.133	−0.19 (−0.27, −0.10)
Model 2	2398	0.134	−0.10 (−0.23, 0.19)
Model 3	2289	0.137	−0.09 (−0.22, 0.04)
Crown–heel length			
Unadjusted	3277	0.025	−0.55 (−0.66, −0.43)
Model 1	2375	0.145	−0.42 (−0.56, −0.28)
Model 2	2373	0.146	−0.31 (−0.50, −0.11)
Model 3	2266	0.148	−0.28 (−0.48, −0.07)

Live singleton births, gestational age ≥37 weeks.

Model 1: Adjusted for maternal education, maternal age, parity, sex of baby, maternal BMI, maternal height, maternal alcohol intake.

Model 2: Adjusted as for Model 1 + maternal smoking (yes/no).

Model 3: Adjusted as for Model 2 + partner smoking (yes/no).

Univariate logistic regression analysis showed no association across tertiles of maternal B‐Cd with decreasing odds of LBW (Table 3), but there was evidence of an association when preterm births were included (Table S5). There was no evidence for a univariate association between maternal B‐Cd and preterm delivery in the whole group (Table S6). Adjustment to the univariate models (models 1–3) did not show evidence for any associations between maternal B‐Cd and the odds of LBW in the group with term deliveries only (Table 3) or in the group with all births (Table S6).

**Table 3:** Associations of tertiles of maternal B‐Cd with birth outcomes in ALSPAC (logistic regressions)

	*n*	*R* ^2^ a	OR (95% CI)			*P* for trend
			T1	T2	T3	
LBW						
Unadjusted	3788	0.001	Ref	1.34 (0.72, 2.51)	0.78 (0.45, 1.37)	0.348
Model 1	2709	0.009	Ref	1.37 (0.66, 2.82)	0.93 (0.66, 2.82)	0.856
Model 2	2707	0.010	Ref	1.38 (0.67, 2.89)	1.39 (0.54, 3.60)	0.399
Model 3	2580	0.009	Ref	1.36 (0.66, 2.80)	0.93 (0.46. 1.89)	0.414

Live singleton births, gestational age ≥37 weeks.

Model 1: Adjusted for maternal age, maternal education, parity, sex of baby, maternal BMI, maternal height, maternal alcohol intake.

Model 2: Adjusted as for Model 1 + maternal smoking (yes/no).

Model 3: Adjusted as for Model 2 + partner smoking (yes/no).

a Cox and Snell pseudo *R* ^2.^

### Regression models for B‐Cd and birth outcomes stratified by smoking status

Repetition of all the models for mothers stratified into smokers and non‐smokers did not provide any evidence for any associations of maternal B‐Cd with birth outcomes in non‐smokers. In smokers there was evidence of a weak adverse association with birthweight for the group with term births only and for the group including all gestational ages that was not evident in non‐smokers (Tables S7 and S8).

### Mediation of Cd in the association between smoking status and birth outcomes

There were strong associations between maternal smoking and anthropometric outcomes (birthweight, head circumference, crown–heel length) in adjusted linear regression models (Table 4) for the main group with no preterm births. The unadjusted models predicted decreases of 171 g in birthweight, 3 mm in head circumference, and 6 mm in crown–heel length for smokers compared with non‐smokers. These associations were not greatly attenuated when maternal B‐Cd was included in the models for birthweight and head circumference, indicating that maternal B‐Cd was not a strong mediator in the association between smoking and anthropometric birth outcomes, although for crown–heel length the association was more attenuated with the inclusion of B‐Cd in the model. There were no associations between smoking and LBW. The results in the whole group including all gestational ages were similar (Table S9).

**Table 4:** Mediation of maternal B‐Cd in associations between smoking and birth outcomes in ALSPAC

	*n*	*R* ^2a^	
Birthweight (g)			Unstandardized B coefficient (95% CI)
Model without adjustment for Cd	2578	0.148	−171.2 (−221.0, −121.0)
Model with adjustment for Cd	2578	0.150	−108.9 (−175.6, −42.2)
Head circumference (cm)			
Model without adjustment for Cd	2289	0.137	−0.30 (−0.45, −0.16)
Model with adjustment for Cd	2289	0.137	−0.21 (−0.41, −0.02)
Crown–heel length (cm)			
Model without adjustment for Cd	2266	0.146	−0.59 (−0.82, −0.35)
Model with adjustment for Cd	2266	0.148	−0.31 (−0.62, 0.01)
LBW			OR (95% CI)
Model without adjustment for Cd	2578	0.010	1.61 (0.77, 3.39)
Model with adjustment for Cd	2578	0.010	1.66 (0.62, 4.39)

Live singleton births, gestational age ≥37 weeks.

Smoking (exposure) (yes/no), birth outcome (outcome), maternal Cd (mediator).

Models adjusted for maternal age, maternal education, sex of baby, parity, maternal BMI, maternal height, maternal alcohol intake, maternal smoking (yes/no), paternal smoking (yes/no).

Cox and Snell pseudo *R* ^2^.

### Sex‐specific effects of maternal B‐Cd on birth outcomes

On stratification by sex, birthweight, head circumference, and crown–heel length were significantly adversely associated with maternal B‐Cd in girls but not in boys in adjusted models (Table 5). The interaction term sex × maternal B‐Cd was significant for all anthropometric variables. The results in the whole group were similar (Table S10)

**Table 5:** Sex‐specific differences in birth outcomes in association with maternal B‐Cd levels in ALSPAC

	*N*	*R* ^2a^	Unstandardized B coefficient (95% CI)	*P* for interaction sex × maternal B‐Cd
Birthweight (g)					
Girls	1274	0.131	−87.1 (−144.8, −29.4)	<0.001
Boys	1304	0.143	−30.8 (−98.6, 37.0)	
Head circumference (cm)				
Girls	1151	0.067	−0.22 (−0.39, −0.04)	<0.001
Boys	1138	0.081	0.05 (−0.14, 0.24)	
Crown–heel length (cm)				
Girls	1141	0.132	−0.44 (−0.71, −0.18)	<0.001
Boys	1125	0.118	−0.07 (−0.38, 0.25)	

Live singleton births, gestational age ≥37 weeks.

Models adjusted for maternal age, maternal education, parity, maternal BMI, maternal height, maternal alcohol intake, maternal smoking (yes/no), paternal smoking (yes/no).

Cox and Snell pseudo *R* ^2^.

## Comments

There was evidence of associations between moderate maternal B‐Cd level and some birth outcomes (birthweight, crown–heel length) but not with others (head circumference, low birthweight) in adjusted regression models in a large cohort of women. However, on stratification by sex there were significant adverse associations between maternal B‐Cd and birthweight, head circumference, and crown–heel length in girls but not in boys. Although maternal B‐Cd was associated linearly with an increase in the number of cigarettes smoked per day, with an additional effect of partner smoking, in univariate analysis, there was no evidence that B‐Cd levels in either smokers or in non‐smokers were associated with adverse birth outcomes. There was no evidence that maternal B‐Cd mediated adverse associations between smoking and birth outcomes.

The mechanisms for the putative effects of Cd on birth outcomes may be related directly to toxic effects on the fetus and/or mediated through toxic effects within the placenta. Although the placenta may act as a partial barrier to Cd being transferred to the fetus, it is likely that at least some Cd accumulates in the placenta.^26^ There it can act as a barrier to the transfer of micronutrients, particularly zinc, by induction of metallothionein production,^3^ and this could restrict fetal growth directly. Cd may also have oestrogenic endocrine disrupting effects,^4^ which could restrict the expression of placental genes linked to growth and development (for example, *11β‐HSD2* and *3β‐HSD*, which control the transfer of glucocorticoids to the fetus^27^). Finally, Cd can act as an epigenic modifier.^28^ These mechanisms suggest that Cd could influence long‐term child developmental outcomes.

Despite these theoretical effects, the evidence from epidemiological studies for negative associations of maternal Cd status with birth outcomes, particularly for outcomes other than birthweight, is mixed. With regard to birthweight, the results of studies of associations with maternal or cord blood Cd and have been conflicting. In the largest study to date, Wang *et al*.^29^ found an adverse association of maternal serum Cd with the baby being SGA in 3254 non‐smoking mother–infant pairs in China, with mothers with serum Cd ≥1.06 μg/L being 43% more likely to have an SGA infant than those with levels <1.06 μg/L in adjusted models. Other studies have found adverse associations of maternal B‐Cd with SGA and/or birthweight in models adjusted for smoking.^14^, ^15^, ^16^ Conversely, other studies have found no associations^7^, ^9^, ^12^, ^30^ or even beneficial associations^31^ with birthweight or SGA in models adjusted or stratified for smoking, even when mean or median maternal B‐Cd levels were high (>1 μg/L).^10^, ^32^, ^33^ There is greater consensus for other birth outcomes (birth length or crown–heel length, head circumference, gestational age, preterm birth), with most studies finding no associations (see Table S5). Studies using tissues other than maternal blood or cord blood to assess Cd status, such as urine, hair or placental tissue are rather more difficult to interpret, but findings are broadly similar, with most studies finding no associations with birth outcomes (see Table S1). Where they exist, differences in the findings of studies could be accounted for, for example, by differences in the confounders that the models include, between‐country differences in health services, or differences in the way in which data on smoking were collected or categorized. Measurement of blood or urine cotinine may be a useful way of measuring smoking exposure in this context^15^ in future studies. In addition, some studies on maternal and/or cord blood Cd levels and birth outcomes have been limited by a small sample size (see Table S1).

To our knowledge, only one previous study has stratified the data by smoking status: Menai *et al*.^34^ found an adverse association of maternal B‐Cd with birthweight in smokers but not in non‐smokers, in accordance with our findings. This could be interpreted as a differential effect reflecting higher Cd levels in smokers.

It is possible that associations of birth outcomes with maternal B‐Cd in mixed‐sex groups could be masked by opposing effects in female and male offspring. It has been suggested that associations, particularly with anthropometric variables, tend to be adverse in girls but beneficial in boys, as was suggested in the present study for head circumference and crown–heel length. This was first proposed by Kippler *et al*.^20^, ^21^ in a cohort in Bangladesh and has subsequently been replicated in cohorts from South Africa and the USA.^8^, ^19^ Kippler *et al*.^20^ suggested several mechanisms for this differential effect: (1) the relative sensitivity of female fetuses compared with male fetuses to a Cd‐induced increase in glucocorticoid concentrations reflecting a decrease in *11β‐HSD2* activity, with a consequent increase in cortisol level; (2) differential Cd interference with the IGF axis, as there is some evidence that levels of IGF subtypes are sex‐specific; (3) the male skeleton may be more resistant to Cd‐induced bone damage through differential effects on bone mineralization, bone density, and bone mineral content; (4) there may be effects on *in utero* sex‐specific programming that contribute to the vulnerability of the fetus to environmental stressors. As suggested by Rollin *et al*.^8^ these effects may be related to hypomethylation of growth‐related genes in girls relative to hypermethylation in boys in response to Cd exposure.^35^

This study has a number of strengths. (1) The study involved a large number of pregnant women with both prenatal B‐Cd and birth outcomes. (2) The prenatal exposure was B‐Cd measured in the first half of pregnancy, in contrast to studies that have relied on cord blood Cd or Cd in other matrices such as hair or urine. (3) The data on maternal smoking were well characterized. (4) We were able to include data on passive smoking. (5) We were able to differentiate effects in female offspring from male offspring. There are some limitations to the study. (1) Although we were able to account for many possible confounders in our analyses, there are likely to be others that were unable to adjust for. These include the effects of other environmental pollutants, such as polychlorinated biphenols.^26^ (2) There were a large number of values for B‐Cd below the limit of detection, making the use of the measurement as a continuous variable in linear models less robust. However, we used a well documented means of correcting values below the limit of detection.

## Conclusion

In accordance with putative mechanisms of the toxic effects of Cd on birth outcomes, we found evidence for independent associations of maternal B‐Cd level with birthweight and crown–heel length in adjusted linear regression models, but not with head circumference. There was no evidence of an increased odds of having a low birthweight baby in adjusted logistic regression models. When the models were repeated after stratification into smokers and non‐smokers, there was evidence for an adverse association with birthweight in smokers. Maternal B‐Cd was not a strong mediator in the associations between smoking and any of the pregnancy outcomes. However, there was strong evidence of differential effects in female and male offspring on anthropometric variables (significant adverse associations of maternal B‐Cd with birthweight, head circumference, and crown–heel length in girls but not in boys). We conclude that sex‐specific effects require further investigation as a possible contributor to the lack of associations found in most mixed‐sex studies.

## Supporting information


**Figure S1.** Flow chart for study.
**Table S1.** Summary of studies on maternal Cd status and birth outcomes.
**Table S2.** Characteristics of pregnant women in ALSPAC included in the present study (singleton livebirth, not preterm).
**Table S3.** Effect of active and passive smoking on maternal B‐Cd levels (μg/L) in ALSPAC.
**Table S4.** Birth outcomes by maternal B‐Cd tertile in ALSPAC.
**Table S5.** Associations of maternal B‐Cd with birth outcomes in ALSPAC (linear regressions).
**Table S6.** Associations of tertiles of maternal B‐Cd with birth outcomes in ALSPAC (logistic regressions).
**Table S7.** Linear regression of maternal B‐Cd with birth outcomes stratified by smoking in ALSPAC.
**Table S8.** Logistic regression for tertiles of maternal B‐Cd with birth outcomes stratified by smoking in ALSPAC.
**Table S9.** Mediation of maternal B‐Cd in associations between smoking and birth outcomes in ALSPAC.
**Table S10.** Sex‐specific differences in birth outcomes in association with maternal B‐Cd levels in ALSPAC.
