# Supplementary material for: Moderate Prenatal Cadmium Exposure and Adverse Birth Outcomes: a Role for Sex‐Specific Differences?
Source: Paediatr Perinat Epidemiol. 2016 Oct 25;30(6):603–11. doi: 10.1111/ppe.12318 (PMC5111596; doi:10.1111/ppe.12318)
Supplement: Supplementary file 1 — Figure S1. Flow chart for study. Table S1. Summary of studies on maternal Cd status and birth outcomes. Table S2. Characteristics of pregnant women in ALSPAC included in the present study (singleton livebirth, not preterm). Table S3. Effect of active and passive smoking on maternal B‐Cd levels (μg/L) in ALSPAC. Table S4. Birth outcomes by maternal B‐Cd tertile in ALSPAC. Table S5. Associations of maternal B‐Cd with birth outcomes in ALSPAC (linear regressions). Table S6. Associations of tertiles of maternal B‐Cd with birth outcomes in ALSPAC (logistic regressions). Table S7. Linear regression of maternal B‐Cd with birth outcomes stratified by smoking in ALSPAC. Table S8. Logistic regression for tertiles of maternal B‐Cd with birth outcomes stratified by smoking in ALSPAC. Table S9. Mediation of maternal B‐Cd in associations between smoking and birth outcomes in ALSPAC. Table S10. Sex‐specific differences in birth outcomes in association with maternal B‐Cd levels in ALSPAC. [file PPE-30-603-s001.docx]

Singleton live births not preterm: n=3828

Blood Cd measured: n=4286

Singletons: n=4209

Singleton live births: n=4191

Preterm (no/yes): n=3828/207 (5.1%)

LBW (no/yes): n=3820/173 (4.3%)

LBW: not preterm (no/yes) n=3719/69 (1.8%)

Neonatal deaths <7 days (no/yes): n=4180/11 (0.3%)

Excluded n=77

Multiples n=61

No delivery details n=1

Missing twin data n=15

Excluded: not live birth n=18

Fetal loss <20 weeks: n=4

Fetal death/stillbirth >20 weeks: n=14

Pregnant women enrolled: n=14,541

**Supplementary Figure 1**

**Supplementary Table 1** Summary of studies on maternal Cd status and birth outcomes

| **Exposure measure** | **Reference** | **Sample size** | **Location** | **Exposure level (µg/l)** | **Above or below reference value^b^** | **Adjusted for maternal smoking** | **Associations** | | | |
| --- | --- | --- | --- | --- | --- | --- | --- | --- | --- | --- |
|  |  |  |  |  |  |  | **Birth weight** | **Birth length/CHL** | **Head circumference** | **Gestational age/preterm** |
| Maternal blood | Wang et al. (2016)^1^ | 3254 | China | Median 0.79 (range 0.04-8.08) (serum) | Below | All non-smokers | Adverse association with SGA | - | - | - |
|  | Bloom et al. (2015)^2^ | 235 | USA | 0.24 (SD 0.14) | Below | Yes | +ve association | No association | No association | No association |
|  | Rollin et al. (2015)^3^ ^c^ | 641 | South Africa | GM 0.25 [95% CI 0.23, 0.27] | Below | Yes | No association | No association | No association | - |
|  | Thomas et al. (2015)^4^ | 1835 | Canada | Median 0.20 | Below | Yes | No association with SGA | - | - | - |
|  | Hu et al. (2015)^5 a^ | 81 | China | NR | - | NR | No association | - | - | - |
|  | Vidal et al. (2015)^6^ | 319 | North Carolina, USA | NR | - | Yes | -ve association | - | - | - |
|  | Al-Saleh et al. (2014)^7 c^ | 1578 | Saudi Arabia | 0.99 (SD 0.31) | Below | All non-smokers | No association | No association | No association | No association |
|  | Johnston et al. (2014)^8^ | 1027 | North Carolina, USA | 0.46 (SD 0.34) | Below | Yes | -ve association  Adverse association with SGA | No association | No association | No association |
|  | Sun et al. (2014)^9^ | 209 | China | 0.60 [95% CI 0.54, 0.66] | Below | Yes | -ve association | No association | - | - |
|  | Ikeh-Tawari et al. (2013)^10^ | 125 | Nigeria | 28.1 (SD 22.5) (serum) | [Above] | No | -ve correlation | -ve correlation | -ve correlation | - |
|  | Menai et al. (2012)^11^ | 901 | France | 0.88 (SD 0.59) | Below | (Stratification) | No association in non-smokers  -ve association in smokers | - | - | - |
|  | Lin et al.(2011)^12 c^ | 289 | Taiwan | 1.05 | Above | All non-smokers | No association | No association | No association | - |
|  | Tian et al. (2009)^13^ | 109 | China | Median 1.80 (0.43-24.24 | Above | All non-smokers | No association | No association | - | - |
|  | Zhang et al. (2004)^14 c^ | 44 | China | Range 0.80-25.50 | [Above] | NR | No association | No association | - | No association |
|  | Nishijo et al. (2004)^15^ | 55 | Japan | 1.04 (SD 0.71), range 0.16-4.45 | Above | (No) | -ve association | -ve association | - | - |
|  |  |  |  |  |  |  |  |  |  |  |
|  | Salpietro et al. (2002)^16a c^ | 45 | Italy | NR | - | All non-smokers | -ve association | - | - | - |
|  | Odland et al. (1999)^17 c^ | 248 114 | Russia  Norway | Median 0.25, range 0.06-3.96  Median 0.20, range 0.06-3.02 | Below | Yes | No association | - | - | - |
| Cord blood | Tang et al. (2016)^18^ | 103 | China | Median 6.36 (IQR 3.36, 13.34) (serum) | - | All non-smokers | No association | No association | No association | No association |
|  | Al-Saleh et al. (2015)^19^ |  |  |  |  |  |  |  |  |  |
|  | Hu et al. (2015)^5 a^ | 81 | China | NR | - | NR | No association | - | - | - |
|  | Rollin et al. (2015)^3 c^ | 641 | South Africa | GM 0.27 [95% CI 0.26-0.29] | - | Yes | -ve association in females only | No association | No association | - |
|  | Zheng et al. (2014)^20^ | 1106 | China | Controls: 0.43 (SD 0.44)  Cases: 0.42 (SD 0.20) | - | NR | - | - | - | No association |
|  | Sun et al. (2014)^9^ | 209 | China | 0.28 [95% CI 0.22, 0.34] | - | Yes | -ve association  Adverse association with SGA | No association | - | - |
|  | Al-Saleh et al. (2014)^7 c^ | 1578 | Saudi Arabia | 0.78 (SD 0.62) | - | All non-smokers | No association | -ve association | No association | No association |
|  | Garcia-Esquinas et al. (2013)^21^ |  | Spain | 0.53 | Below | Yes | No association | No association | - | - |
|  | Lin et al. (2011)^12 c^ | 289 | Taiwan | 0.31 | Below | All non-smokers | No association | No association | -ve association | - |
|  | Tian et al. (2009)^13^ | 109 | China | Median 0.60 (range 0.02-1.78) | - | All non-smokers | -ve association | -ve association | - | - |
|  | Zhang et al. (2004)^14a c^ | 44 | China | NR | - | NR | No association | -ve association | - | No association |
|  | Salpietro et al. (2002)^16 c^ | 45 | Italy | NR | - | All non-smokers | -ve association | - | - | - |
|  | Odland et al. (1999)^17 c^ | 98 124 | Russia  Norway | Median 0.06, range 0.06-0.54  Median 0.06, range 0.06-4.34 | - | Yes | No association | - | - | - |
|  | Galicia-Garcia et al. (1997)^22^ | 49 | Mexico | 1.4 (SD 0.4) | Above | Yes | No association | - | - | - |
| Placenta | Al-Saleh et al. (2015)^19^ |  |  |  |  |  |  |  |  |  |
|  | Xu et al. (2015)^23^ | 262 | China | Recycling area: median 96.8 ng/g  Reference area: median 20.9 ng/g | - | Yes | -ve association | - | - | - |
|  | Al-Saleh et al. (2014)^7^ | 1578 | Saudi Arabia | 0.06 (SD 0.40) µg/g dry wt | - | All non-smokers | No association | No association | No association | No association |
|  | Tian et al. (2009)^13^ | 109 | China | Median 0.15 (range 0.03-3.97) µg/g dry wt | - | All non-smokers | - | - | - | - |
|  | Llanos and Ronco (2009)^24^ | 20 | Chile | Control: 0.023 (SD 0.002) µg/g/ dry wt Fetal growth restriction: 0.050 (SD 0.006) | - | All non-smokers | -ve association | - | - | - |
|  | Zhang et al. (2004)^14^ | 44 | China | 0.08-3.97 µg/g dry wt | - | NR | No association | No association | - | No association |
|  | Falcon et al. (2003)^25 a^ | NR | NR | NR |  | All smokers | -ve association | -ve association | -ve association | -ve association |
|  | Frery et al. (1993)^26 a^ | 102 | NR | NR | - | Yes | No association | - | - | - |
|  | Fagher et al. (1993)^27 a^ | 30 | Poland Sweden | 0.3 µg/g 0.1 µg/g | - | Yes | - | - | - | No association |
|  | Loiacono et al. (1992)^28^ | 161 | NR | NR | - | All non-smokers | No association | - | - | - |
|  | Berlin et al. (1992)^29^ | 266 | NR | 0.021 (SD 0.022), range <0.002-0.095 µg/g | - | NR | No association | - | - | - |
| Urine | Romano et al. (2016)^30^ | 396 | USA | GM 0.31 µg/g creatinine | - | Yes | No association | -ve association in girls  +ve association in boys | No association | - |
|  | Rollin et al. (2015)^3^ | 641 | South Africa | GM 0.29 [95% CI 0.27, 0.31] (creatinine-corrected) | - | Yes | No association | No association | No association | - |
|  | Bloom et al. (2014)^2^ | 235 | USA |  | - | Yes | - | - | - | - |
|  | Sun et al. (2014)^9^ | 209 | China | 0.92 [95% CI 0.52, 1.29] | - | Yes | No association | No association | - | - |
|  | Kippler et al. (2012)^31^ | 1616 | Bangladesh | 0.09 (SD 0.01) µg/l (adjusted to mean specific gravity of samples) | - | All non-smokers | -ve association in girls | No association | -ve association in girls | - |
|  | Shirai et al. (2010)^32^ | 78 | Japan | 0.98 (SD 0.89), range <0.04-7.29 µg/g creatinine |  | Yes | -ve association | No association | No association | - |
|  | Nishijo et al. (2002)^33^ | 57 | Japan | NR | - | Yes | - | - | - | -ve association |
| Hair | Frery et al. (1993)^26^ ^a^ | 102 | France | NR | - | Yes | -ve association | - | - | - |

Values for exposure levels are means unless stated otherwise.

NR, not reported.

^a^Abstract only.

^b^Schulz et al. (2007)^34^: 1 µg/l.

^c^Data on both maternal and cord B-Cd.

**References for Supplementary Table 1**

1. Wang H, Liu L, Hu Y-F, Hao J-H, Chen Y-H, Su P-Y, et al. Maternal serum cadmium level during pregnancy and its association with small for gestational age infants: a population-based birth cohort study. *Scientific Reports*. 2016; 6:22631.

2. Bloom MS, Buck Louis GM, Sundaram R, Maisog JM, Steuerwald AJ, Parsons PJ. Birth outcomes and background exposures to select elements, the Longitudinal Investigation of Fertility and the Environment (LIFE). *Environ Res*. 2015; 138:118-129.

3. Rollin HB, Kootbodien T, Channa K, Odland JO. Prenatal Exposure to Cadmium, Placental Permeability and Birth Outcomes in Coastal Populations of South Africa. *PLoS One*. 2015; 10:e0142455.

4. Thomas S, Arbuckle TE, Fisher M, Fraser WD, Ettinger A, King W. Metals exposure and risk of small-for-gestational age birth in a Canadian birth cohort: The MIREC study. *Environ Res*. 2015; 140:430-439.

5. Hu X, Zheng T, Cheng Y, Holford T, Lin S, Leaderer B, et al. Distributions of heavy metals in maternal and cord blood and the association with infant birth weight in China. *J Reprod Med*. 2015; 60:21-29.

6. Vidal AC, Semenova V, Darrah T, Vengosh A, Huang Z, King K, et al. Maternal cadmium, iron and zinc levels, DNA methylation and birth weight. *BMC Pharmacol Toxicol*. 2015; 16:20.

7. Al-Saleh I, Shinwari N, Mashhour A, Rabah A. Birth outcome measures and maternal exposure to heavy metals (lead, cadmium and mercury) in Saudi Arabian population. *Int J Hyg Environ Health*. 2014; 217:205-218.

8. Johnston JE, Valentiner E, Maxson P, Miranda ML, Fry RC. Maternal cadmium levels during pregnancy associated with lower birth weight in infants in a North Carolina cohort. *PLoS One*. 2014; 9:e109661.

9. Sun H, Chen W, Wang D, Jin Y, Chen X, Xu Y. The effects of prenatal exposure to low-level cadmium, lead and selenium on birth outcomes. *Chemosphere*. 2014; 108:33-39.

10. Ikeh-Tawari EP, Anetor JI, Charles-Davies MA. Cadmium level in pregnancy, influence on neonatal birth weight and possible amelioration by some essential trace elements. *Toxicol Int*. 2013; 20:108-112.

11. Menai M, Heude B, Slama R, Forhan A, Sahuquillo J, Charles MA, et al. Association between maternal blood cadmium during pregnancy and birth weight and the risk of fetal growth restriction: the EDEN mother-child cohort study. *Reproductive Toxicology*. 2012; 34:622-627.

12. Lin CM, Doyle P, Wang D, Hwang YH, Chen PC. Does prenatal cadmium exposure affect fetal and child growth? *Occup Environ Med*. 2011; 68:641-646.

13. Tian LL, Zhao YC, Wang XC, Gu JL, Sun ZJ, Zhang YL, et al. Effects of gestational cadmium exposure on pregnancy outcome and development in the offspring at age 4.5 years. *Biol Trace Elem Res*. 2009; 132:51-59.

14. Zhang YL, Zhao YC, Wang JX, Zhu HD, Liu QF, Fan YG, et al. Effect of environmental exposure to cadmium on pregnancy outcome and fetal growth: a study on healthy pregnant women in China. *J Environ Sci Health A Tox Hazard Subst Environ Eng*. 2004; 39:2507-2515.

15. Nishijo M, Tawara K, Honda R, Nakagawa H, Tanebe K, Saito S. Relationship between newborn size and mother's blood cadmium levels, Toyama, Japan. *Arch Environ Health*. 2004; 59:22-25.

16. Salpietro CD, Gangemi S, Minciullo PL, Briuglia S, Merlino MV, Stelitano A, et al. Cadmium concentration in maternal and cord blood and infant birth weight: a study on healthy non-smoking women. *J Perinat Med*. 2002; 30:395-399.

17. Odland JO, Nieboer E, Romanova N, Thomassen Y, Lund E. Blood lead and cadmium and birth weight among sub-arctic and arctic populations of Norway and Russia. *Acta Obstet Gynecol Scand*. 1999; 78:852-860.

18. Tang M, Xu C, Lin N, Liu K, Zhang Y, Yu X, et al. Lead, mercury, and cadmium in umbilical cord serum and birth outcomes in Chinese fish consumers. *Chemosphere*. 2016; 148:270-275.

19. Al-Saleh I, Al-Rouqi R, Obsum CA, Shinwari N, Mashhour A, Billedo G, et al. Interaction between cadmium (Cd), selenium (Se) and oxidative stress biomarkers in healthy mothers and its impact on birth anthropometric measures. *Int J Hyg Environ Health*. 2015; 218:66-90.

20. Zheng G, Zhong H, Guo Z, Wu Z, Zhang H, Wang C, et al. Levels of heavy metals and trace elements in umbilical cord blood and the risk of adverse pregnancy outcomes: a population-based study. *Biol Trace Elem Res*. 2014; 160:437-444.

21. Garcia-Esquinas E, Perez-Gomez B, Fernandez-Navarro P, Fernandez MA, de Paz C, Perez-Meixeira AM, et al. Lead, mercury and cadmium in umbilical cord blood and its association with parental epidemiological variables and birth factors. *BMC Public Health*. 2013; 13:841.

22. Galicia-García V, Rojas-Lopez M, Rojas R, Olaiz G, Rios C. Cadmium levels in maternal, cord and newborn blood in Mexico city. *Toxicol Lett*. 1997; 91:57-61.

23. Xu X, Chiung YM, Lu F, Qiu S, Ji M, Huo X. Associations of cadmium, bisphenol A and polychlorinated biphenyl co-exposure in utero with placental gene expression and neonatal outcomes. *Reproductive Toxicology*. 2015; 52:62-70.

24. Llanos MN, Ronco AM. Fetal growth restriction is related to placental levels of cadmium, lead and arsenic but not with antioxidant activities. *Reproductive Toxicology*. 2009; 27:88-92.

25. Falcon M, Vinas P, Perez-Carceles MD, Luna A. Placental cadmium and lipid peroxidation in smoking women related to newborn anthropometric measurements. *Arch Environ Contam Toxicol*. 2003; 45:278-282.

26. Frery N, Nessmann C, Girard F, Lafond J, Moreau T, Blot P, et al. Environmental exposure to cadmium and human birthweight. *Toxicology*. 1993; 79:109-118.

27. Fagher U, Laudanski T, Schutz A, Sipowicz M, Akerlund M. The relationship between cadmium and lead burdens and preterm labor. *Int J Gynaecol Obstet*. 1993; 40:109-114.

28. Loiacono NJ, Graziano JH, Kline JK, Popovac D, Ahmedi X, Gashi E, et al. Placental cadmium and birthweight in women living near a lead smelter. *Arch Environ Health*. 1992; 47:250-255.

29. Berlin M, Blanks R, Catton M, Kazantzis G, Mottet NK, Samiullah Y. Birth weight of children and cadmium accumulation in placentas of female nickel-cadmium (long-life) battery workers. *IARC Sci Publ*. 1992:257-262.

30. Romano ME, Enquobahrie DA, Simpson C, Checkoway H, Williams MA. Maternal body burden of cadmium and offspring size at birth. *Environ Res*. 2016; 147:461-468.

31. Kippler M, Tofail F, Gardner R, Rahman A, Hamadani JD, Bottai M, et al. Maternal cadmium exposure during pregnancy and size at birth: a prospective cohort study. *Environ Health Perspect*. 2012; 120:284-289.

32. Shirai S, Suzuki Y, Yoshinaga J, Mizumoto Y. Maternal exposure to low-level heavy metals during pregnancy and birth size. *J Environ Sci Health A Tox Hazard Subst Environ Eng*. 2010; 45:1468-1474.

33. Nishijo M, Nakagawa H, Honda R, Tanebe K, Saito S, Teranishi H, et al. Effects of maternal exposure to cadmium on pregnancy outcome and breast milk. *Occup Environ Med*. 2002; 59:394-396; discussion 397.

34. Schulz C, Angerer J, Ewers U, Kolossa-Gehring M. The German Human Biomonitoring Commission. *Int J Hyg Environ Health*. 2007; 210:373-382.

**Supplementary Table 2**Characteristics of pregnant women in ALSPAC included in the present study (singleton live birth, not preterm)

|  |  |
| --- | --- |
| Age (years) | 28.0±4.90 (n=3638) |
| Parity |  |
| 0 | 1553 (40.6%) |
| ≥1 | 1983 (51.8%) |
| BMI (kg/m^2^) | 23.00±3.77 (n=3264) |
| Education |  |
| None/CSE/Vocational | 984 (28.4%) |
| O level | 1154 (33.3%) |
| A level/degree | 1328 (38.3%) |
| Gestation length | 39.8±1.31 (n=3828) |
| Smoking (no cigarettes per days) |  |
| 0 | 2804 (73.2%) |
| 1–5 | 200 (5.2%) |
| 6–10 | 251 (6.6%) |
| 11–15 | 145 (3.8%) |
| ≥16 | 127 (3.3%) |
| Partner smokes |  |
| Yes | 1317 (37.8%) |
| No | 2166 (62.2%) |
| Alcohol (measures per week) |  |
| 0 | 2340 (69.1%) |
| 1–10 | 934 (27.6%) |
| 11–20 | 70 (2.1%) |
| ≥21 | 44 (1.3%) |

Live singleton births, gestational age ≥37 weeks.

Values are n (%) or mean±SD.

**Supplementary Table 3**Effect of active and passive smoking on maternal B-Cd levels (µg/l) in ALSPAC

|  | **Mean±SD** | **P value** |
| --- | --- | --- |
| Maternal B-Cd by no. cigarettes smoked per day^a^ |  | <0.001 |
| 0 | 0.32±0.31 (n=2804) |  |
| 1–5 | 1.03±0.55 (n=200) |  |
| 6–10 | 1.35±0.66 (n=251) |  |
| 11–15 | 1.56±0.71 (n=145) |  |
| ≥15 | 1.75±0.65 (n=127) |  |
|  |  |  |
| Maternal B-Cd by active and passive smoking |  | <0.001 |
| Mother smoker, partner smoker | 1.39±0.69 (n=552) |  |
| Mother smoker, partner non-smoker | 1.34±0.67 (n=171) |  |
| Mother non-smoker, partner smoker | 0.37±0.33 (n=693) |  |
| Mother non-smoker, partner non-smoker | 0.29±0.27 (n=1899) |  |

Live singleton births, gestational age ≥37 weeks.

^a^Mean B-Cd of smokers 1.37±0.67 µg/l, n=723; non-smokers 0.32±0.31, n=2804.

**Supplementary Table 4**Birth outcomes by maternal B-Cd tertile in ALSPAC

|  | **Tertile of maternal B-Cd** | | | **P value** |
| --- | --- | --- | --- | --- |
|  | **T1** | **T2** | **T3** |  |
|  |  |  |  |  |
| Mean maternal B-Cd (µg/l) | 0.16±0.03  (range 0.14-0.22, n=1441) | 0.31±0.06  (range 0.23-0.46, n=1352) | 1.26±0.67  (range 0.47-6.30, n=1398) |  |
|  |  |  |  |  |
| Birthweight (g) | 3489.2±543.0, n=1403 | 3474.0±539.9, n=1299 | 3310.1±560.7, n=1291 | <0.001 |
| Head circumference (cm) | 34.90±1.52, n=1212 | 34.93±1.40, n=1215 | 34.63±1.51, n=1126 | <0.001 |
| Crown–heel length (cm) | 50.92±2.26, n=1194 | 50.98±2.28, n=1110 | 50.31±2.33, n=1113 | <0.001 |
| Preterm |  |  |  |  |
| Yes | 69 (4.9%) | 62 (4.7%) | 76 (5.8%) | 0.376 |
| No | 1349 (95.1%) | 1251 (95.3%) | 1288 (94.2%) |  |
| LBW |  |  |  |  |
| Yes | 54 (3.8%) | 44 (3.4%) | 75 (5.9%) | 0.004 |
| No | 1349 (96.2%) | 1268 (96.6%) | 1203 (94.1%) |  |
|  |  |  |  |  |

Live singleton births, all gestational ages.

**Supplementary Table 5**Associations of maternal B-Cd with birth outcomes in ALSPAC (linear regressions)

|  | **n** | **R^2^** | **Unstandardised B coefficient [95% CI]** | **p** |
| --- | --- | --- | --- | --- |
|  |  |  |  |  |
| Birthweight |  |  |  |  |
| Unadjusted | 3993 | 0.024 | -139.3 [-166.9, -111.8] | <0.001 |
| Model 1 | 2836 | 0.130 | -121.2 [-154.3, -88.1] | <0.001 |
| Model 2 | 2834 | 0.133 | -66.9 [-112.4, -21.3] | 0.004 |
| Model 3 | 2696 | 0.131 | -60.0 [-107.8, -12.10] | 0.014 |
| Head circumference |  |  |  |  |
| Unadjusted | 3463 | 0.007 | -0.20 [-0.28, -0.21] | <0.001 |
| Model 1 | 2504 | 0.109 | -0.17 [-0.27, -0.08] | <0.001 |
| Model 2 | 2502 | 0.111 | -0.07 [-0.2, 0.07] | 0.331 |
| Model 3 | 2384 | 0.109 | -0.06 [-0.20, 0.08] | 0.385 |
| Crown–heel length |  |  |  |  |
| Unadjusted | 3417 | 0.020 | -0.53 [-0.65, -0.41] | <0.001 |
| Model 1 | 2472 | 0.128 | -0.44 [-0.59, -0.29] | <0.001 |
| Model 2 | 2470 | 0.131 | -0.23 [-0.44, -0.03] | 0.027 |
| Model 3 | 2354 | 0.133 | -0.22 [-0.43, -0.01] | 0.045 |
| Model 3 with additional adjustment for gestational age |  |  |  |  |
| Birthweight | 2696 | 0.363 | -39.0 [-80.0, 2.07] | 0.063 |
| Head circumference | 2384 | 0.310 | -0.36 [-0.16, 0.09] | 0.563 |
| Crown–heel length | 2354 | 0.305 | -0.18 [-0.37, 0.02] | 0.073 |
|  |  |  |  |  |

Live singleton births, all gestational ages.

Model 1: Adjusted for maternal education, age, parity, sex of baby, maternal BMI, maternal height, maternal alcohol intake

Model 2: Adjusted as for Model 1 + maternal smoking (yes/no)

Model 3: Adjusted as for Model 2 + partner smoking (yes/no)

**Supplementary Table 6**Associations of tertiles of maternal B-Cd with birth outcomes in ALSPAC (logistic regressions)

|  | **n** | **R^2^** ^a^ | **OR [95% CI]** | | | **P for trend** |
| --- | --- | --- | --- | --- | --- | --- |
|  |  |  | **T1** | **T2** | **T3** |  |
|  |  |  |  |  |  |  |
| Preterm |  |  |  |  |  |  |
| Unadjusted | 4035 | 0.000 | Ref | 1.03 [0.73, 1.46], p=0.860 | 0.83 [0.59, 1.16], p=0.265 | 0.264 |
| Model 1 | 2864 | 0.006 | Ref | 1.31 [0.84, 2.05], p=0.228 | 0.99 [0.64, 1.52], p=0.956 | 0.953 |
| Model 2 | 2862 | 0.007 | Ref | 1.33 [0.85, 2.08], p=0.206 | 1.40 [0.75, 2.60], p=0.291 | 0.184 |
| Model 3 | 2724 | 0.008 | Ref | 1.37 [0.75, 2.68], p=0.178 | 1.42 [0.75, 2.68], p=0.282 | 0.167 |
| LBW |  |  |  |  |  |  |
| Unadjusted | 3993 | 0.003 | Ref | 1.14 [0.76, 1.71], p=0.552 | 0.65 [0.45, 0.93], p=0.018 | 0.015 |
| Model 1 | 2836 | 0.014 | Ref | 1.62 [0.95, 2.79], p=0.076 | 0.80 [0.50, 1.28], p=0.357 | 0.411 |
| Model 2 | 2834 | 0.016 | Ref | 1.66 [0.97, 2.85], p=0.066 | 1.28 [0.65, 2.50], p=0.475 | 0.230 |
| Model 3 | 2696 | 0.015 | Ref | 1.67 [1.00, 2.90], p=0.067 | 1.28 [0.64, 2.53], p=0.486 | 0.231 |
| Model 3 with additional adjustment for gestational age |  |  |  |  |  |  |
| Preterm^b^ | 2724 | 0.302 | Ref | - | - | - |
| LBW | 2696 | 0.110 | Ref | 1.59 [0.83, 3.05], p=0.163 | 1.33 [0.58, 3.05], p=0.495 | 0.298 |

Live singleton births, all gestational ages.

Model 1: Adjusted for maternal education, parity, sex of baby, maternal BMI, maternal height, maternal alcohol intake

Model 2: Adjusted as for Model 1 + maternal smoking (yes/no)

Model 3: Adjusted as for Model 2 + partner smoking (yes/no)

^a^Cox and Snell pseudo R^2^

^b^Model failed to converge.

**Supplementary Table 7**Linear regression of maternal B-Cd with birth outcomes stratified by smoking in ALSPAC

|  | **All gestational ages** | | | | **Gestational age ≥37 weeks** | | | |
| --- | --- | --- | --- | --- | --- | --- | --- | --- |
|  | **n** | **R^2^** | **B [95% CI]** | **p** | **n** | **R^2^** | **B [95% CI]** | **p** |
|  |  |  |  |  |  |  |  |  |
| **Smokers** |  |  |  |  |  |  |  |  |
| Birthweight (g) |  |  |  |  |  |  |  |  |
| Unadjusted | 765 | 0.007 | -66.18 [-123.60, -8.76] | <0.001 | 718 | 0.014 | -82.06 [-321.76, 31.35] | 0.002 |
| Model 1 | 538 | 0.093 | -63.03 [-127.05, 1.00] | 0.054 | 505 | 0.131 | -62.57 [-119.97, -5.18] | 0.033 |
| Model 2 | 486 | 0.094 | -62.18 [-128.48, 4.12] | 0.066 | 459 | 0.122 | -60.92 ]-121.79, -0.05] | 0.050 |
| Head circumference (cm) |  |  |  |  |  |  |  |  |
| Unadjusted | 662 | 0.001 | -0.07 [-0.23, 0.09] | 0.394 | 630 | 0.004 | -0.13 [0.28, 0.02] | 0.096 |
| Model 1 | 472 | 0.112 | -0.02 [-0.20, 0.16] | 0.819 | 448 | 0.135 | -0.07 [-0.24, 0.10] | 0.401 |
| Model 2 | 426 | 0.117 | -0.04 [-0.23, 0.14] | 0.658 | 408 | 0.136 | -0.08 [-0.26, 0.09] | 0.351 |
| Crown–heel length (cm) |  |  |  |  |  |  |  |  |
| Unadjusted | 653 | 0.003 | -0.19 [-0.45, 0.07] | 0.158 | 621 | 0.010 | -0.31 [-0.55, -0.07] | 0.012 |
| Model 1 | 467 | 0.114 | -0.11 [-0.41, 0.19] | 0.462 | 443 | 0.160 | -0.23 [-0.49, 0.039] | 0.095 |
| Model 2 | 421 | 0.123 | -0.10 [-0.41, 0.20] | 0.506 | 403 | 0.159 | -0.20 [-0.48, 0.08] | 0.153 |
| Model 2 with additional adjustment for gestational age | | | | |  |  |  |  |
| Birthweight (g) | 486 | 0.381 | -43.37 [-98.31, 11.56] | 0.121 |  |  |  |  |
| Head circumference (cm) | 426 | 0.348 | -0.06 [-0.22, 0.10] | 0.447 |  |  |  |  |
| Crown–heel length (cm) | 421 | 0.360 | -0.14 [-0.40, 0.12] | 0.294 |  |  |  |  |
| **Non-smokers** |  |  |  |  |  |  |  |  |
| Birthweight (g) |  |  |  |  |  |  |  |  |
| Unadjusted | 2901 | 0.001 | -1.56 [-114.74, 12.96] | 0.118 | 2774 | 0.001 | -51.88 [-109.59, 5.82] | 0.078 |
| Model 1 | 2296 | 0.125 | -58.02 [-126.25, 10.21] | 0.096 | 2202 | 0.140 | -67.50 [-130.55, -4.45] | 0.036 |
| Model 2 | 2210 | 0.125 | -40.00 [-111.55, 33.56] | 0.292 | 2219 | 0.139 | -45.52 [-112.65, 21.61] | 0.184 |
| Head circumference (cm) |  |  |  |  |  |  |  |  |
| Unadjusted | 2530 | 0.001 | -0.13 [-0.32, 0.06] | 0.180 | 2428 | 0.000 | -0.09 [-0.26, 0.07] | 0.272 |
| Model 1 | 2030 | 0.107 | -0.09 [-0.29, 0.11] | 0.389 | 1950 | 0.129 | -0.10 [-0.9, 0.08] | 0.269 |
| Model 2 | 1958 | 0.109 | -0.05 [-0.26, 0.16] | 0.651 | 1881 | 0.132 | -0.06 [-0.25, 0.14] | 0.576 |
| Crown–heel length (cm) |  |  |  |  |  |  |  |  |
| Unadjusted | 2496 | 0.002 | -0.31 [-0.59, -0.02] | 0.035 | 2404 | 0.002 | -0.31 [-0.58, -0.05] | 0.022 |
| Model 1 | 2003 | 0.125 | -0.33 [-0.63, -0.12] | 0.037 | 1930 | 0.134 | -0.34 [-0.63, -0.04] | 0.026 |
| Model 2 | 1933 | 0.129 | -0.29 [-0.61, 0.03] | 0.125 | 1863 | 0.137 | -0.30 [-0.60, 0.01] | 0.060 |
| Model 2 with additional adjustment for gestational age | | | |  |  |  |  |  |
| Birthweight (g) | 2210 | 0.348 | -16.67 [-79.35, 46.02] | 0.602 |  |  |  |  |
| Head circumference (cm) | 1958 | 0.299 | 0.03 [-0.16, 0.21] | 0.777 |  |  |  |  |
| Crown–heel length (cm) | 1933 | 0.288 | -0.17 [-0.46, 0.12] | 0.248 |  |  |  |  |

Live singleton births.

Model 1: Adjusted for maternal education, parity, sex of baby, maternal BMI, maternal height, maternal alcohol intake.

Model 2: Adjusted as for Model 1 + partner smoking (yes/no).

**Supplementary Table 8**Logistic regression for tertiles of maternal B-Cd with birth outcomes stratified by smoking in ALSPAC

|  | **All gestational ages** | | | | | **Gestational age ≥37 weeks** | | | | |
| --- | --- | --- | --- | --- | --- | --- | --- | --- | --- | --- |
|  | **OR [95% CI]** | | | **n** | **P for trend** | **OR [95% CI]** | | | **n** | **P for trend** |
|  | **T1** | **T2** | **T3** |  |  | **T1** | **T2** | **T3** |  |  |
| **Non-smokers** |  |  |  |  |  |  |  |  |  |  |
| Preterm |  |  |  |  |  |  |  |  |  |  |
| Unadjusted | Ref ^a^ | - | - |  |  |  |  |  |  |  |
| Model 1 | Ref | 1.17 [0.80, 1.70], p=0.427 | 1.43 [0.79, 2.58], p=0.233 | 2933 | 0.198 |  |  |  |  |  |
| Model 2 | Ref | 1.31 [0.84, 2.05], p=0.228 | 0.99 [0.64, 1.52], p=0.956 | 2864 | 0.953 |  |  |  |  |  |
| Model 3 | Ref ^a^ | - | - | 2726 |  |  |  |  |  |  |
| LBW |  |  |  |  |  |  |  |  |  |  |
| Unadjusted | Ref ^a^ | - | - |  |  | Ref ^a^ | - | - |  |  |
| Model 1 | Ref | 1.31 [0.85, 2.03], p=0.227 | 0.96 [0.54, 1.71], p=0.895 | 2933 | 0.715 | Ref ^a^ | - | - |  |  |
| Model 2 | Ref | 1.62 [0.95, 2.79], p=0.076 | 0.80 [0.51, 1.28], p=0.357 | 2836 | 0.411 | Ref ^a^ | - | - |  |  |
| Model 2 with additional adjustment for gestational age | | | | | |  |  |  |  |  |
| Preterm | Ref ^a^ | - | - | 2726 |  |  |  |  |  |  |
| LBW | Ref | 1.56 [0.82, 3.00], p=0.177 | 0.89 [0.48, 0.65], p=0.713 | 2698 | 0.970 |  |  |  |  |  |
| **Smokers** |  |  |  |  |  |  |  |  |  |  |
| Preterm |  |  |  |  |  |  |  |  |  |  |
| Unadjusted ^a^ | Ref ^a^ | - | - |  |  |  |  |  |  |  |
| Model 1^a^ | Ref ^a^ | - | - |  |  |  |  |  |  |  |
| Model 2^a^ | Ref ^a^ | - | - |  |  |  |  |  |  |  |
| LBW |  |  |  |  |  |  |  |  |  |  |
| Unadjusted ^a^ | Ref ^a^ | - | - |  |  | Ref ^a^ | - | - |  |  |
| Model 1 ^a^ | Ref ^a^ | - | - |  |  | Ref ^a^ | - | - |  |  |
| Model 2 ^a^ | Ref ^a^ | - | - |  |  | Ref ^a^ | - | - |  |  |
| Model 2 with additional adjustment for gestational age | | | |  |  |  | - | - |  |  |
| Preterm | Ref ^a^ | - | - |  |  |  |  |  |  |  |
| LBW | Ref ^a^ | - | - |  |  |  |  |  |  |  |

Live singleton births.

Model 1: Adjusted for maternal education, parity, sex of baby, maternal BMI, maternal height, maternal alcohol intake.

Model 2: Adjusted as for Model 1 + partner smoking.

^a^Model failed to converge.

**Supplementary Table 9 Mediation of maternal B-Cd in associations between smoking and birth outcomes in ALSPAC**

|  | **n** | **R^2^** |  | **P value** |
| --- | --- | --- | --- | --- |
|  |  |  | **Unstandardised B coefficient [95% CI]** |  |
| Birthweight (g) |  |  |  |  |
| Model without adjustment for Cd | 2696 | 0.130 | -177.0 [-231.2, -122.9] | <0.001 |
| Model with adjustment for Cd | 2696 | 0.131 | -117.3 [-189.4, -45.1] | 0.001 |
| Head circumference (cm) |  |  |  |  |
| Model without adjustment for Cd | 2384 | 0.112 | -0.29 [-0.45, -0.14] | <0.001 |
| Model with adjustment for Cd | 2384 | 0.109 | -0.23 [-0.44, -0.02] | 0.029 |
| Crown–heel length (cm) |  |  |  |  |
| Model without adjustment for Cd | 2354 | 0.132 | -0.64 [-0.89, -0.40] | <0.001 |
| Model with adjustment for Cd | 2354 | 0.133 | -0.42 [-0.75, -0.09] | 0.012 |
|  |  |  |  |  |
| LBW |  |  | **OR [95% CI]** |  |
| Model without adjustment for Cd | 2696 | 0.014 | 1.73 [1.03, 2.91] | 0.039 |
| Model with adjustment for Cd | 2696 | 0.014 | 1.68 [0.84, 3.34] | 0.143 |
| Preterm |  |  |  |  |
| Model without adjustment for Cd^a^ | 2724 | 0.007 | 1.30 [0.79, 2.12] | 0.305 |
| Model with adjustment for Cd^a^ | 2724 | 0.007 | 1.41 [0.58, 1.47] | 0.320 |
|  |  |  |  |  |

Live singleton births, all gestational ages.

Smoking (exposure) (yes/no), birth outcome (outcome), maternal Cd (mediator).

Models adjusted for maternal age, maternal education, parity, maternal BMI, maternal height, maternal alcohol intake, maternal smoking (yes/no), paternal smoking (yes/no).

^a^Cox and Snell pseudo R^2^.

**Supplementary Table 10 Sex-specific differences in birth outcomes in association with maternal B-Cd levels in ALSPAC**

|  | **n** | **R^2^** ^a^ |  | **P value** | **P for interaction sex × maternal B-Cd** |
| --- | --- | --- | --- | --- | --- |
|  |  |  | **Unstandardised B coefficient [95% CI]** |  |  |
| Birthweight (g) |  |  |  |  |  |
| Girls | 1319 | 0.122 | -80.87 [-143.17, -18.58] | 0.011 | <0.001 |
| Boys | 1377 | 0.127 | -35.17 [-108.32, 37.98] | 0.346 |  |
| HC (cm) |  |  |  |  |  |
| Girls | 1189 | 0.057 | -0.20 [-0.39, -0.01] | 0.036 | <0.001 |
| Boys | 1195 | 0.073 | 0.07 [-0.11, 0.30] | 0.363 |  |
| CHL (cm) |  |  |  |  |  |
| Girls | 1176 | 0.131 | -0.41 [-0.68, -0.13] | 0.004 | <0.001 |
| Boys | 1178 | 0.106 | 0.01 [-0.33, 0.34] | 0.965 |  |
|  |  |  |  |  |  |

Live singleton births, all gestational ages.

Models adjusted for maternal age, maternal education, parity, maternal BMI, maternal height, maternal alcohol intake, maternal smoking (yes/no), paternal smoking (yes/no).

^a^Cox and Snell pseudo R^2^.
